# Health Risks from Intake and Contact with Toxic Metal-Contaminated Water from Pager River, Uganda

**DOI:** 10.3390/jox13040035

**Published:** 2023-09-26

**Authors:** Patrick Onen, Robin Akemkwene, Caroline K. Nakiguli, Daniel Nimusiima, Daniel Hendry Ruma, Alice V. Khanakwa, Christopher Angiro, Gadson Bamanya, Boniface Opio, Allan Gonzaga, Timothy Omara

**Affiliations:** 1 Department of Chemistry, University of Kerala, Thiruvananthapuram 695581, India; 2 Department of Chemistry, Faculty of Education and Humanities, Gulu University, Gulu P.O. Box 166, Uganda; 3 Department of Chemistry, Faculty of Science, Mbarara University of Science and Technology, Mbarara P.O. Box 1410, Uganda; 4 Department of Nutritional Sciences and Dietetics, Kyambogo University, Kampala P.O. Box 1, Uganda; 5 Department of Environmental Health and Disease Prevention, Faculty of Public Health, Lira University, Lira P.O. Box 1035, Uganda; 6 School of Water, Energy and Environment, Water Science Institute, Cranfield University, College Road, Cranfield MK43 0AL, UK; 7 Department of Physical Sciences, Kampala International University, Kampala P.O. Box 20000, Uganda; 8 Department of Science and Vocational Education, Lira University, Lira P.O. Box 1035, Uganda; 9 Chemistry Division, Testing Department, Uganda National Bureau of Standards, Kampala P.O. Box 6329, Uganda

**Keywords:** average daily dose, cancer risk, estimated daily intake, target hazard quotient, toxic metals

## Abstract

Pollution of water resources is one of the major impediments to the realization of Sustainable Development Goals, especially in developing countries. The aim of this study was to investigate the physicochemical quality and potentially toxic element (lead and cadmium) concentrations in surface water sampled from Pager River, a tributary of the Nile River in Northern Uganda. Water samples (*n* = 18) were taken from six different points upstream (A, B, and C) and downstream (D, E, and F) of the river and analyzed following standard methods for their physiochemical properties. Atomic absorption spectroscopy was used to quantify lead and cadmium concentrations. Human health risks from ingestion and dermal contact with potentially toxic metal-contaminated water were calculated. The results obtained indicated that the mean temperature (27.7 ± 0.5–29.5 ± 0.8 °C), turbidity (40.7 ± 2.1–50.1 ± 1.1 NTU), lead (0.296 ± 0.030–0.576 ± 0.163 mg/L) and cadmium (0.278 ± 0.040–0.524 ± 0.040 mg/L) occurred at levels that surpassed their permissible limits as per World Health Organization guidelines for drinking water. Human health risk assessment showed that there are potential non-cancer risks from the ingestion of water from Pager River by adults, as the total hazard quotients were greater than one. These results emphasize the urgency to restrict the dumping of wastes into the river to minimize chances of impacting the Nile River, which flows northwards to the Mediterranean Sea. Further studies should perform routine monitoring of the river during both dry and wet seasons to establish the spatiotemporal variations of physicochemical, microbial, and trace metal profiles of the river and the associated health risks.

## 1. Introduction

Water is one of the most important and infinite resources with an increasingly growing demand. It is a fundamental resource for life as well as economic development [1,2]. In the simplest definition, water is life [3], but its availability is becoming compromised by the increasing global population, resource-intensive economic development, and the introduction of anthropogenic contaminants [4]. One of the “Global Goals”, i.e., the sixth Sustainable Development Goal, emphasizes maintenance of water quality and availability. However, deterioration of water quality by inorganic, legacy, and emerging organic pollutants has led to water insecurity, nutrient–climate synergized eutrophication, and harmful algal blooms [5,6]. Thus, the protection and regulation of water quality only transcend national boundaries in the developed countries, i.e., in developing countries, water pollution is less regulated [4]. 

Africa has one of the largest numbers of water resources. The continent has some of the most unique freshwater systems worldwide [7,8]. Eastern Africa is among the most environmentally diverse regions of Africa, and this diversity is reflected in its hydrology, characterized by various water resources such as Lake Victoria, Lake Tanganyika, Lake Malawi, and the Nile River [9]. Contamination of water resources in the region by both legacy and emerging organic and inorganic contaminants has been reported [10]. Of these, contamination by potentially toxic elements (PTEs) has been the most pronounced, and it is one of the critical parameters being monitored in drinking water in the region [11]. PTEs, previously termed as heavy metals, are elements with high molecular weights (or densities that are at least five-fold greater than that of water) [12]. They include lead (Pb), cadmium (Cd), copper, iron, zinc, nickel, vanadium, arsenic, chromium, and mercury, among others [13]. These PTEs are toxic, especially at higher concentrations, but their levels of toxicity are influenced by valence states, exposure routes and times, bioavailable forms and levels, and the ingested quantity [14]. 

Lead (density = 11.34 g/cm^3^) is a relatively unreactive post-transition element that is known to be neurotoxic [15,16]. Long-term exposure causes Pb poisoning and hypertension [17,18]. According to available statistics from the World Health Organization (WHO), exposure to Pb causes at least 900,000 deaths per year and up to 30% of the global burden of developmental intellectual disability [19]. Inorganic Pb compounds are also listed as probable human carcinogens (Group 2A) [20]. Although there has been global compliance with the United States Environmental Protection Agency (US EPA) 1991 Lead and Copper Rule and the ban on the use of leaded fuel, drinking water remains an important source of exposure to Pb [21]. Thus, the WHO has enlisted Pb as a priority chemical hazard that should be rigorously monitored in drinking water [19]. Cadmium (density = 8.65 g/cm^3^), on the other hand, is a silvery element implicated in the etiology of hypertension, hepatic injury, malignancies, kidney dysfunction, and arteriosclerosis [22]. Short-term exposure to high levels of Cd can induce vomiting, diarrhea, coughing, chest pain, muscle cramps, loss of dental alveola, osteotoxicity, salivation, and sensory imbalances [23,24]. Similar to Pb, Cd is classified as a probable human carcinogen (Group B1) by the US EPA and International Agency for Research on cancer [25]. It is, therefore, one of the 10 WHO priority elements that has to be monitored in drinking water [19]. 

This study considered the Pager River, one of the rivers in Uganda. According to a recent study [26], the physicochemical and microbiological parameters of water in the Pager River are above the regulatory limits set by the Uganda National Environmental Management Authority. The river is majorly polluted by dumped wastes [27], and anecdotal reports have positioned that there are some illnesses due to using water from the Pager River [28]. In 2021, the Pager and Akeca Rivers were reportedly polluted by a strange whitish chemical that caused fish, lizard, and tortoise deaths [29]. The aim of this study was, therefore, to investigate the physicochemical quality and Pb and Cd concentrations in water sampled from the Pager River, a tributary of the Nile River in Northern Uganda. The human health risk assessment model proposed by the US EPA [30] was used to establish potential health risks that could be experienced by the local population after dermal contact and drinking water from the river.

## 2. Materials and Methods

### 2.1. Study Area

**Figure 1:**
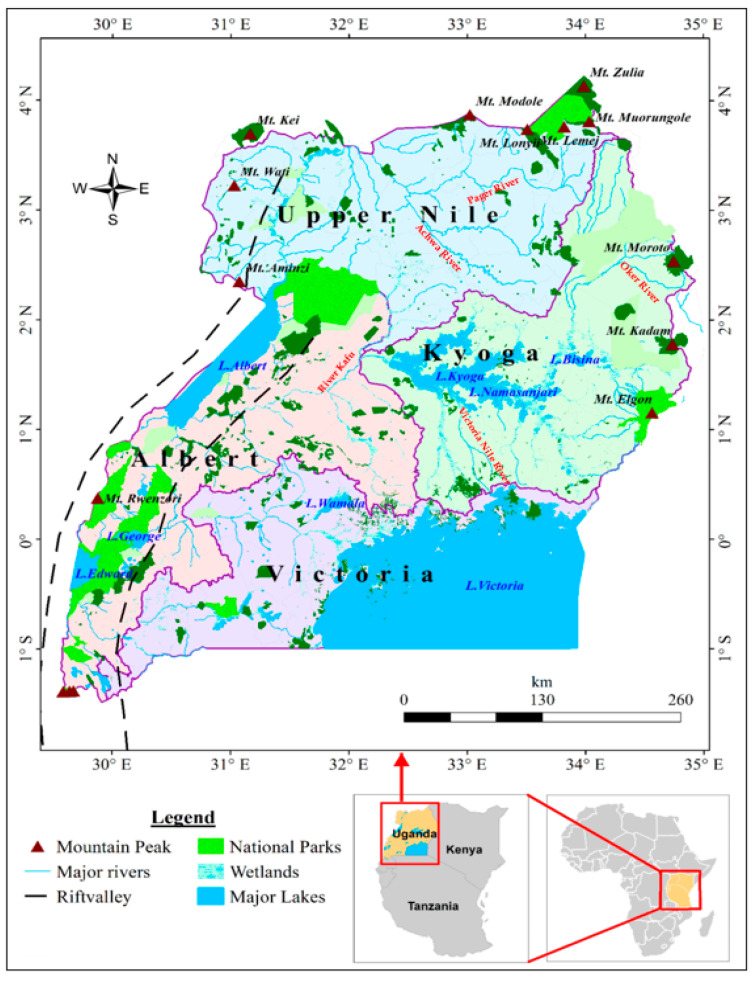
Map showing the location of the Pager River and some other major rivers in Uganda. Adapted from Onyutha et al. [42].

The Pager River (latitude: 3°16′2.28″, longitude: 33°5′29.4″) is the longest perennial river and tributary of the Nile River, the longest river in Africa. The river is located in the Kitgum District (“the place of good luck”), Northern Uganda, East Africa at an elevation of 973 m above sea level (Figure 1) [31]. It flows through the sub-counties of Labongo Layamo, Labongo Akwang, and Labongo Amida, Kitgum municipality and joins the Achwa River with the Agago River after covering a distance of 194.79 km [32,33,34,35]. The Pager River is part of the Achwa River basin, and it was chosen in this study because: (i) its banks have dense settlements (Kitgum Matidi), agricultural farm lands, and car-washing bays that are prone to flooding after its banks burst [28,31,36,37]; (ii) its location in one of the three districts that suffered the brunt of the Lord’s Resistance Army civil wars in Northern Uganda (between 1986 and 2006) and therefore had the highest number of internally displaced persons in camps [38]; and (iii) it covers both the nodding syndrome and onchocerciasis-hyperendemic foci that is in proximity to blackfly breeding sites. Along with the Aswa and Agago Rivers, the Pager River was aerially sprayed in 2012 to eradicate blackflies in the region [39,40]. 

### 2.2. Chemicals and Reagents Used

Deionized water (conductivity < 1.0 µS/cm, with undetectable Pb and Cd contents) was from the Uganda National Bureau of Standards (UNBS), Chemistry Laboratory, Kampala, Uganda. All the other reagents and chemicals were of high analytical purity and were supplied by Merck (Darmstadt, Germany), Sigma-Aldrich (St. Louis, MO, USA), or HiMedia Pvt Laboratories (Mumbai, India).

### 2.3. Sampling Procedures 

To characterize water quality and its spatial variability along the river network, two different sampling sites were chosen to study the physiochemical parameters of the Pager River. These sites were the Guu A Parish and the Westland A Parish. The Guu A Parish is where the river approaches the Kitgum municipality and is characterized by activities such as cattle bathing, cloth laundering, and agriculture. It includes the northern pediment slopes of the flat-topped granitic Pandwong inselberg, which is encircled on all its sides by the Pager River [31]. Westland A Parish, on the other hand, is located at the center of the Kitgum municipality (next to the Kitgum main market, with over 500 salespersons) and is the most densely populated part of the district, with grass-thatched houses (slums) and very poor sanitation, drainage, and waste-disposal practices [27,41]. It has small-scale industries and local brewers who discharge untreated industrial wastes into the Pager River [27]. 

A total of eighteen (18) water samples were collected from six sites designated as A, B, and C in the Guu A Parish (upstream) and D, E, and F in the Westland A parish (downstream). Sampling was performed in June 2023 at a depth of 10–15 cm below the water surface using clean 500 mL polyethylene terephthalate bottles with screw caps.

### 2.4. Physiochemical Analysis of Samples

Non-conservable parameters (temperature, pH, electrical conductivity, and total dissolved solids) of the samples were measured onsite using an HI 98129-HI 98130 pH/TDS/temperature/conductivity meter (Hanna Instruments Inc., Woonsocket, RI, USA). Turbidity was measured using a calibrated Palintest photometer 7100 (Palintest^®^, Gateshead Tyne & Wear, UK) following the manufacturer’s instructions.

### 2.5. Lead and Cadmium Contents of the Water Samples

The collected samples from each site were evaporated to dryness. Concentrated nitric acid (10 mL), perchloric acid (2 mL), and hydrofluoric acid (4 mL) were added to the residue and then heated to dryness once more. The final residue was thereafter reconstituted in 2 mL of 2M hydrochloric acid, transferred into a 25 mL volumetric flask, and topped to the mark with deionized water. The solution was then analyzed for PTEs (Pb and Cd) using a double-beam, microcomputer-controlled Perkin-Elmer 2380 atomic absorption spectrophotometer (Perkin Elmer Inc., Waltham, MA, USA). These were achieved at wavelengths of 283.3 nm for Pb and 228.8 nm for Cd. The PTEs’ concentrations were obtained from regression equations of calibration curves prepared using absorbances of working concentrations diluted from 1000 mg/L stock solutions of Pb nitrate and Cd chloride.

### 2.6. Human Health Risk Assessment Due to PTEs Intake and Dermal Contact

For human health risks associated with PTEs, the assessment is performed by taking into consideration the potential cancer and non-cancer health effects in adults (“as the general population”) and children (“as a sensitive group”). For non-carcinogenic health risks, the average daily doses are computed to cater for the direct intake of water (DIT*_water_*) and dermal contact (DC*_water_*) (Equations (1) and (2)) [43,44].

(1) $$DITwater=\frac{PTEC\times W_{IG}\times E_{FQ}\times E_{DT}}{W_{B}\times T_{AE}}$$

(2) $$DCwater=\frac{HCM\times SAF\times SDAF\times AF\times E_{FQ}\times E_{DT}}{W_{B}\times T_{AE}}\times 10^{-6}$$

where *PTEC* = *PTE*’s concentration in water (mg/L); *W_IG_* (water intake rate; L/day) = 1.8 and 21 for children and adults; *E_FQ_* = exposure frequency (365 days/year); *E_DT_* = exposure time, equal to the average lifetime (58.65 years) for Ugandans [44,45]; *W_B_ =* average body weight (=15 kg for children and 60 kg for adults); *T_AE_* = average exposure time, expressed as *E_FQ_ × E_DT_* [46]; *SAF* is the exposed dermal surface area = 2800 and 24,350 cm^2^ for children and adults [43]; DAFC is the dermal absorption factor = 0.01 for carcinogenic metals [47]; and *SADF* is the skin adherence factor = 0.2 and 0.7 mg/cm^2^/day for children and adults [48].

To assess non-carcinogenic health risks, the target hazard quotient (THQ) is calculated (Equations (3) and (4)). In consequence, THQ ≤ 1 shows unlikely occurrence of adverse health effects in an exposed individual and vice versa [44,48]. Because such effects are augmentative for PTEs, the cumulative health risks per exposure pathway (total THQ _DIT*water*_ and total THQ _DC*water*_) may be calculated (Equations (5) and (6)) [30].

(3) $$THQ\;DITwater=\frac{{DIT}_{water}}{R_{f}DIT}$$

(4) $$THQ\;DCwater=\frac{{DC}_{water\;}}{R_{f}DC}$$

(5) $$Total\;THQ\;DITwater=\sum \frac{{DIT}_{water}}{R_{f}DIT\;}$$

(6) $$Total\;THQ\;DCwater=\sum \frac{{DC}_{water\;}}{R_{f}DC}$$

From these, *R_f_DIT* is the oral reference dose, while *R_f_DC* is the dermal reference dose, with established toxicological values [30]. A reference dose of a potentially toxic element is defined as the highest amount of it which, when ingested through a given pathway, may not result in the development of deleterious health effects by an individual in their lifetime [49]. 

Cancer risks for PTEs that are probable carcinogens (CRV) are usually estimated as the incremental lifetime cancer risk (Equation (7)). The total risk values (TCRV) can also be calculated (Equation (8)) by considering the cumulative toxic effects of PTEs [47,50].

CRV = DIT*_water_* × ICSF (7)

(8) $$TCRV=\sum_{i=1}^{n}CRV\;$$

where the ingestion cancer slope factor (ICSF) represents the contaminant-specific risk generated by an average lifetime amount of 1 mg/kg/day of the carcinogenic, potentially toxic element [47]. 

### 2.7. Quality Assurance and Quality Control of Data

All experiments were performed in triplicate. For PTE analyses, linearities of the calibration curves were checked, and these were within acceptable limits (R^2^ > 0.995 in all cases). Further, the analytical quality of absorbances obtained was guaranteed through the analysis of blanks and spikes, whose recoveries (range: 97–101%) were analytically considered acceptable. Relative standard deviations of the experiments (analytical precision) ranged between 3.9% and 4.7%.

### 2.8. Data Analysis

Experimental results data from triplicate analyses were entered into Excel, averaged, and tabulated as the mean ± standard deviation of replicates. Statistically significant differences in the physicochemical quality of water among the sampling sites on the Pager River were evaluated using one-way analysis of variance with Tukey’s test. Pearson’s bivariate correlation and principal component analysis (PCA) were used to explore the inter-relationships between PTE concentrations and the physicochemical parameters of the samples. The analyses were executed at a 95% confidence interval, employing GraphPad Prism for Windows (version 9, GraphPad Software, San Diego, CA, USA).

## 3. Results

### 3.1. Physiochemical Parameters of the Water Samples

The physiochemical properties of water samples from the Pager River, Northern Uganda are shown in Table 1. The temperature (27.7 ± 0.5–29.5 ± 0.8 °C) and turbidity (40.7 ± 2.1–50.1 ± 1.1 NTU) recorded were outside the recommended guidelines provided by the WHO for potable water [51]. 

### 3.2. PTE Contents of the Water Samples

**Figure 2:**
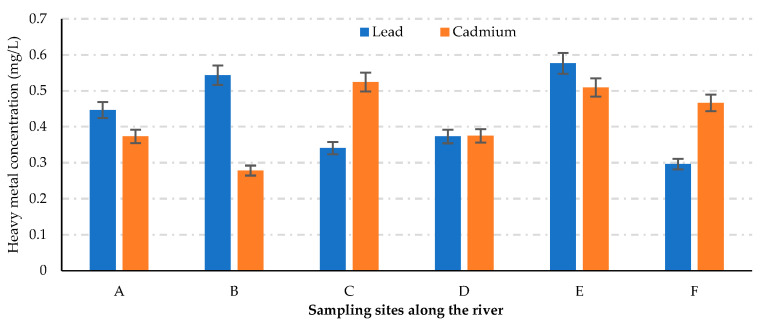
Concentrations of lead and cadmium along the sampled stretch of the Pager River, Northern Uganda. Sites A, B, and C are in the Guu A Parish (upstream), while D, E, and F are in the Westland A parish (downstream).

In this study, Pb and Cd were quantified in water samples taken from the Pager River, Uganda (Figure 2). They occurred at mean concentrations of 0.296 ± 0.030–0.576 ± 0.163 mg/L and 0.278 ± 0.040–0.524 ± 0.040 mg/L, respectively. Higher metal concentrations were recorded downstream (sites D, E, and F). These values surpassed the recommended WHO limits of 0.01 and 0.003 mg/L for Pb and Cd in drinking water [51].

### 3.3. Interrelationships between Physicochemical Parameters and PTEs 

**Figure 3:**
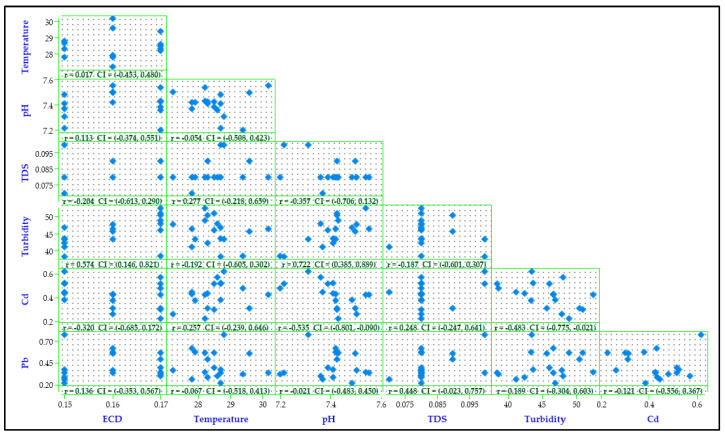
Pearson’s correlation matrix plot for the physicochemical parameters and PTEs of water from the sampled stretch of the Pager River, Northern Uganda.

**Figure 4:**
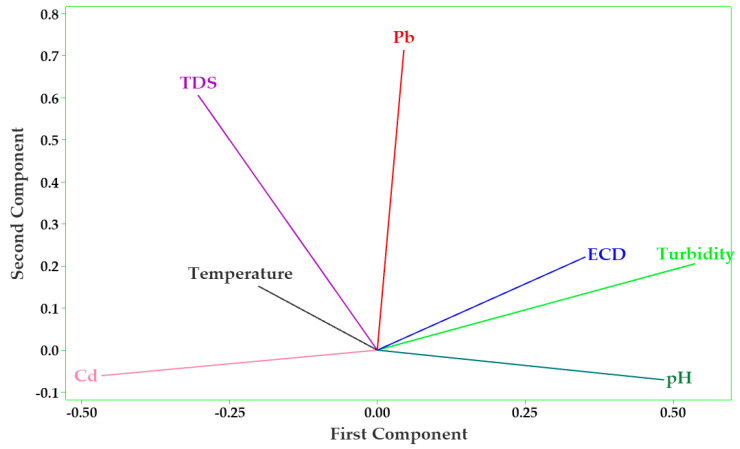
PCA loading plot showing the effect of physicochemical parameters influencing the variation of PTEs of water from the sampled stretch of the Pager River, Northern Uganda.

As shown in the Pearson’s correlation matrix plot (Figure 3), most of the parameters had weak and insignificant negative correlations (Supplementary materials: Table S1). Weak but significant negative correlations were observed between Cd and turbidity (r = −0.483, *p* = 0.042), as well as Cd and pH (r = −0.535, *p* = 0.022). A negative correlation was also observed between Pb and Cd concentrations (r = −0.121, *p* = 0.633). However, turbidity and pH (r = 0.722, *p* = 0.001), turbidity and ECD (r = 0.574, *p* = 0.013), Pb and TDS (r = 0.448, *p* = 0.062), and Cd and TDS (r = 0.248, *p* = 0.320) had positive correlations. These results agreed well with PCA results (Figure 4). With consideration of eigenvalues greater than 1 (for *p* < 0.05), the first three principal components explained up to 73.6% of the total variance observed. However, major correlations could only be observed in the first principal component (Supplementary materials: Table S2). 

### 3.4. Human Health Risks from Intake and Dermal Contact with Contaminated Water 

The calculated daily doses for the direct intake of contaminated water were from 3.34 × 10^−2^ mg/kg/day for Cd from point B by children to 2.01 × 10^−1^ mg/kg/day for Pb in water at point E drunk by adults (Table 2). These values were higher than the oral reference doses for the metals in the case of adults. The (total) target hazard quotients were greater than 1 for all the sampled points (Table 3), and Cd was the major contributor to these effects.

For dermal contact, the daily doses spanned from 1.03 × 10^−7^ mg/L/day for Cd ingested by children in water from point B to 1.64 × 10^−6^ mg/L/day for Pb in water at point E consumed by adults. These values were all lower than the dermal reference doses of Pb and Cd. As shown by the calculated THQ and TTHQ values (Table 3), no deleterious health effects are likely to be experience by individuals who come into dermal contact with water contaminated with Pb and Cd at the sampled stations of the river.

Contrastingly, the potential cancer risks calculated (Table 4) spanned from 0.0009 × 10^−4^ for Pb at point F for water drunk by adults to 0.68 × 10^−4^ for Pb in water at point E drunk by adults. In all cases, the cancer risk values as well as the total (cumulative) cancer risk values fell within the US EPA permissible limit of 10^−6^–10^−4^ [47]. 

## 4. Discussion

### 4.1. Physicochemical Profile of Water Samples

Temperature is the degree of hotness or coldness of a substance [52]. It affects the physical, chemical, and microbiological processes in water bodies (including the flowing water in rivers). It is an essential parameter used to evaluate the quality of drinking water, and the WHO limit is 15 °C, which was surpassed in this study. In reference to a previous study in the Turag River (Bangladesh), a similarly high temperature was found (23.9–31.2 °C) [53]. Such high temperatures may alter the color, viscosity, solubility, and taste of water [54,55,56], thereby reducing its palatability. 

Similarly, the hydrogen potential (pH) of water measured in this study fell outside the acceptable range of the WHO. Undoubtingly, pH affects several parameters of water, including taste and hardness. Broadly speaking, low pH of water can result in the release of hydrogen sulphide, which is toxic [52]. Corrosion of water pipes also occurs in water with low pH, while high pH values above 9.0 affect the chlorination of water, reduce the concentration of iron, phosphates, and sulphates, and lead to the conversion of carbon dioxide into hydrogen carbonates and carbonates [52,57]. It is positioned that the intake of acidic or alkaline water may be harmful to the body [58]. Moreover, such pH values tend to make water unpleasant, i.e., conferring a bitter or metallic taste [59]. Compared to previous studies, the pH values recorded in our study were comparable to 6.44–8.19 in Uganda’s Manafwa and Nyamwamba Rivers [60,61], 5.85–8.30 in the Rwandese Nyabarongo and Nyabugogo rivers[50], the Mohokare River of Lesotho [62], and the Rwimi, Nyamugasani, and Aturukuku Rivers in Uganda [63,64,65]. These values were higher than the 5.58–6.80 values found in the Mubuku and Nyamwamba Rivers [64]. 

We also measured the electrical conductivity (ECD) of the river water samples. ECD is the measure of electric current flowing through a solution of water due to ions in it. It is related to the TDS, as well as the water’s temperature [51]. The permissible limit of ECD lies in the range of 750 to 1000 µS/cm, as per WHO guidelines on drinking water [51]. In reference to previous reports, the ECD values obtained were lower than 37.9–3780 and 30–150 µS/cm [61] for the River Nyamwamba and River Mubuku, 88.7–122.2 µS/cm (River Manafwa) [60], 118.57 µS/cm (River Sio), 140.82 µS/cm (River Victoria Nile), 80.44 µS/cm (River Lhubiriha), 63.15 and 12–119 µS/cm (River Mobuku), 460.51 µS/cm (River Lubigi), 946.08 µS/cm (River Nyamugasani), 43–103 and 81–220 µS/cm (River Rwimi), 108–1524 µS/cm (River Musamya), and 99.91 µS/cm (River Nyamwamba) of Uganda [63,66,67]. In the Rwandese Nyabarongo and Nyabugogo rivers, ECDs of 74.3–102.0 µS/cm were reported [50]. Other reports for the Mohokare River (Lesotho) [62] and River Rido (Nigeria) [68] showed ECDs of 2000–3800 µS/cm and 79–146.3 µS/cm, which are higher than those obtained in this study.

For turbidity, the values obtained were eight- to ten-fold higher than the value of <5 NTU indicated for potable water. Such turbidity values suggest the presence of pathogens or particles that can shield pathogenic organisms from disinfectants. Lastly, the TDS of the water samples were determined because it affects the taste of water if its value is >2000 mg/L [51,52]. TDS ranged from 0.07 ± 0.00 to 0.10 ± 0.00 mg/L across the sampling sites. Statistically, TDS did not vary significantly among the sampled sites (*p* > 0.05) and were within the recommendable limit of 1000 mg/L [51]. Studies performed on the Mubuku and Nyamwamba Rivers of Uganda [61] and the Jamuna and Turag Rivers of Bangladesh [53,69] found much higher TDS values (1937–6580, 1344.0, 106–131 and 0–1244 mg/L, respectively) than found in this study. 

### 4.2. PTE Concentrations in the Water Samples

In this study, the concentrations of Pb and Cd in water samples from the Pager River, Uganda ranged from 0.278 to 0.576 mg/L. These results were higher than that of Cd and Pb in the Turag River (Bangladesh) [53], the Bolong and Rongna Rivers (China) [70], the Dzindi, Madanzhe, and Mvudi Rivers (South Africa), the Manafwa River and River Mubuku (Uganda) [60,64], the Mara River (Tanzania) [71], and the Tisa River (Romania) [72] (Table 3). Higher Pb and Cd concentrations than found in this study were previously reported in water samples from the Nyamwamba River (Uganda) [61], the Sosiani River (Kenya) [73], the River Kabul (Pakistan) [74], and the Kor River (Iran) [75] (Table 5).

Both Pb and Cd are non-essential metal ions in the human body. In the context of Pb, the high levels recorded in this river may be related to metal fabrication activities and irresponsible disposal of used Pb-based batteries, as well as the continued use of leaded gasoline [76,77]. Thus, exposure to Pb in the water may result in conditions such as kidney failure, high blood pressure, anemia, Pb poisoning, neurotoxicity, cancer, weakness, and brain damage (for higher doses) [15,16,17,18]. On the other hand, the high levels of Cd found in the Pager River may be explained by mobilized runoff water from metal fabrication works, the use of Cd-based phosphate fertilizers, disposed nickel–Cd batteries, and sewage [78]. In terms of toxicity, short-term exposure to such high levels of Cd can induce vomiting, diarrhea, coughing, chest pain, muscle cramps, salivation, sensory imbalances, hepatic injury, and renal failure [23]. Epidemiological data have pointed out that high-level exposure to Cd has been associated with a high risk of developing lung, kidney, and prostate cancers [79].

**Table 5:** Comparison of concentrations (mg/L) of toxic metals in water from the Pager River with previous studies.

River (Country)	Pb	Cd	References
Pager River (Uganda)	0.296–0.576	0.278–0.524	This study
Manafwa River (Uganda)	0.002–0.010	0.001–0.002	Opolot et al. [60]
River Nyamwamba (Uganda)	0.40–8.21	0.05–1.40	Masereka et al. [61]
	0.27–0.40	—	Mwesigye and Tumwebaze [80]
	0.047	—	Mukisa et al. [64]
River Mubuku (Uganda)	0.053	—	
River Rwimi (Uganda)	0.067	—	
Kagera River & tributaries (Rwanda)	0.045	0.965	Nshimiyimana et al. [81]
Mara River (Tanzania)	0.01–0.71	BDL–0.11	Kihampa and Wenaty [71]
Tisa River (Romania)	0.0016–0.00514	0.00011–0.00206	Dippong et al. [72]
Turag River (Bangladesh)	—	0.0068–0.017	Ahmed et al. [53]
Bolong river (China)	0.00034–0.00064	0.00011–0.00054	Luo et al. [70]
Rongna River (China)	0.00049–0.00241	0.00012–0.00064	
Nyabarongo river (Rwanda)	0.05–0.75	BDL-0.106	Omara et al. [50,82]
Nyabugogo river (Rwanda)	0.59	BDL	Omara et al. [50]
Sosiani river (Kenya)	0.02–1.89	—	Amadi [73]
Marimba River (Zimbabwe)	0.213–0.544	—	Mvungi et al. [83]
Dzindi, Mvudi, and Madanzhe Rivers (South Africa)	0.0105–0.0201	0.0016–0.0093	Okonkwo et al. [84]
River Ganga (India)	0.037–0.163	0.001–0.059	Gupta et al. [85]
Akcay River (Turkey)	BDL–0.00036	—	Leventeli and Yalcin [86]
River Kabul (Pakistan)	0.337–0.81	0.015–0.038	Afzaal et al. [74]
Kor River (Iran)	—	0.02–17.36	Mokarram et al. [75]

— means not determined. BDL = Below method detection limit.

### 4.3. Interrelationships between Physicochemical Parameters and PTEs of the Water Samples

The PTEs were not strongly correlated with the water quality parameters determined. In environmental studies, such weak and negative correlations suggest that the parameters do not strongly influence the contamination levels of the PTEs. Specifically, negative correlations between Pb and Cd showed that they originated from different sources and possessed different characteristics and transport behaviors [87,88,89,90], and therefore, any increase or decrease in their concentrations in the river water could only occur disproportionately. On the other hand, turbidity and pH, turbidity and ECD, Pb and TDS, Cd and TDS with positive correlations suggested that the presence of the PTEs in the water positively influenced the levels of these parameters. Multivariate statistical analysis (PCA) indicated that the observed levels of PTEs in the water from the Pager River were due to anthropogenic contributions from different sources. 

### 4.4. Human Health Risk Assessment Results

For non-cancer risks, the computed average daily doses through the intake of contaminated water were higher than the oral reference doses for the PTEs in the case of adults, indicating that there are discernable non-carcinogenic health effects that may be experienced by the general population who drink water from the Pager River. In our previous study on the Manafwa River (Eastern Uganda), we found that non-carcinogenic health effects could be experienced in both children and adults only when water was taken during the dry season [60]. Masereka et al. [61] reported that the total target hazard quotients for water from the Nyamwamba River exceeded 1 for both direct ingestion and dermal contact, supporting the occurrence of several non-carcinogenic health effects reported in the Kilembe Mines region of Western Uganda. These reports underscore the need to examine the health risks of water from the Pager River during both the dry and wet seasons to examine whether the health risks may be higher in the dry season than observed for samples collected in June 2023, which was a wet season. 

For cancer risks, all the individual cancer risk values, as well as the total cancer risk values, were within the safe limit of 1 × 10^−6^ to 1 × 10^−4^. This implies that there are no potential cancer risks from drinking water from the different stretches of the Pager River. Our findings align well with Opolot et al. [60], who found that cancer risk values for Ugandans who drink water from Manafwa River did not suggest any carcinogenic health risks. A study in the Nyamwamba River of Western Uganda, however, found cancer risk values through the ingestion of PTEs to range from 0.119× 10^−6^ for Pb ingested by adults to 7.815 × 10^−1^ for arsenic ingested by children [61], which suggested cancer risks in both age groups. Taken together, the results of the human health risk assessment in our study may not point to the potential occurrence of deleterious health effects, due to the limited number of PTEs studied. Since the effects of PTEs are known to be cumulative, a further study considering a larger number of PTEs in the river water should be undertaken.

## 5. Conclusions and Future Perspectives

In this study, the physicochemical profile, including the Pb and Cd contents of surface water sampled from the Pager River, a tributary of the Nile River in Northern Uganda, was investigated. We found that the mean temperature (27.7 ± 0.5–29.5 ± 0.8 °C), turbidity (40.7 ± 2.1–50.1 ± 1.1 NTU), Pb (0.296 ± 0.030–0.576 ± 0.163 mg/L), and Cd (0.278 ± 0.040–0.524 ± 0.040 mg/L) levels of the water surpassed the compliance limits set by the WHO for drinking water. This could cause adverse health effects, especially in adults, where the computed average daily doses through ingestion of water were higher than the oral reference doses for the metals. The results obtained in our study were lower than those reported in some studies from Uganda and Africa. Further studies should investigate the levels of PTEs in the Pager River during both the dry and wet seasons, so as to establish the potential variations in the PTE concentrations and the associated health risks. Assessment of the concentrations of other contaminants such as pesticides, per- and polyfluoroalkyl substances, active pharmaceutical ingredients, and microplastics should be initiated, since this river was aerially sprayed during onchocerciasis elimination campaigns.

## Figures and Tables

**Table 1:** Physiochemical parameters of water from different points along the Pager River, Uganda.

Parameter ^1^	A	B	C	D	E	F	WHO [51]
pH	7.49 ± 0.06	7.43 ± 0.00	7.32 ± 0.01	7.51 ± 0.04	7.38 ± 0.06	7.33 ± 0.10	6.5–8.5
Electrical conductivity (µS/cm)	0.163 ± 0.006	0.170 ± 0.000	0.170± 0.000	0.157 ± 0.006	0.153 ± 0.006	0.150 ± 0.000	1500
Temperature (°C)	27.7 ± 0.5	28.3 ± 0.5	28.3 ± 0.5	29.5 ± 0.8	28.5 ± 0.5	28.3 ± 0.5	15
Total dissolved solids (mg/L)	0.10 ± 0.00	0.09 ± 0.01	0.09 ± 0.00	0.08 ± 0.00	0.08 ± 0.00	0.07 ± 0.00	1000
Turbidity (NTU)	49.0 ± 3.1	50.1 ± 1.1	42.3 ± 5.0	46.4 ± 0.6	43.5 ± 0.2	40.7 ± 2.1	5.0

^1^ Results are presented as means ± standard deviation of triplicates. Sites A, B, and C are in the Guu A Parish (upstream), while D, E, and F are in the Westland A parish (downstream).

**Table 2:** Average daily doses of water samples from the Pager River, Uganda.

Daily Dose	Age Group	Sampling Point	Pb	Cd
DIT*_water_* (×10^−2^ mg/kg/day)	Children	A	5.35	4.48
		B	6.52	3.34
		C	4.09	6.29
		D	4.48	4.50
		E	6.91	6.11
		F	3.55	5.59
DC*_water_* (×10^−7^ mg/L/day)		A	1.67	1.39
		B	2.03	1.03
		C	1.27	1.96
		D	1.39	1.40
		E	2.15	1.90
		F	1.11	1.74
DIT*_water_* (×10^−1^ mg/kg/day)	Adults	A	1.56	1.31
		B	1.90	0.97
		C	1.19	1.83
		D	1.31	1.31
		E	2.01	1.78
		F	1.04	1.63
DC*_water_* (×10^−6^ mg/L/day)		A	1.27	1.06
		B	1.54	0.79
		C	0.97	1.49
		D	1.06	1.07
		E	1.64	1.45
		F	0.84	1.32
Oral reference dose (mg/kg/day) [47]			1.4 × 10^−1^	1 × 10^−3^
Dermal reference dose (mg/L/day) [30]			5.25 × 10^−4^	6.0 × 10^−5^

Sites A, B, and C are in the Guu A Parish (upstream), while D, E, and F are in the Westland A parish (downstream).

**Table 3:** Target hazard quotients for ingestion and dermal contact with water from the Pager River, Uganda.

Age Group	Pathway	Sampling Point	Target Hazard Quotient		Total Target Hazard Quotient
			Pb	Cd	
Children	Ingestion	A	0.382	44.8	45.182
		B	0.466	33.4	33.866
		C	0.292	62.9	63.192
		D	0.320	45.0	45.320
		E	0.494	61.1	61.594
		F	0.254	55.9	56.154
	Dermal contact	A	0.00032	0.0023	0.00262
		B	0.00039	0.0017	0.00209
		C	0.00024	0.0033	0.0354
		D	0.00026	0.0023	0.00256
		E	0.00041	0.0032	0.00361
		F	0.00021	0.0029	0.00311
Adults	Ingestion	A	1.11	131	132.11
		B	1.36	97	98.36
		C	0.85	183	183.85
		D	0.94	131	131.94
		E	1.44	178	179.44
		F	0.74	163	163.74
	Dermal contact	A	0.0024	0.0177	0.0201
		B	0.0029	0.0132	0.0161
		C	0.0018	0.0248	0.0266
		D	0.0020	0.0178	0.0198
		E	0.0031	0.0241	0.0272
		F	0.0016	0.0220	0.0236

Sites A, B, and C are in the Guu A Parish (upstream), while D, E, and F are in the Westland A parish (downstream).

**Table 4:** Cancer risk values (×10^−4^) for drinking water from the Pager River, Uganda.

Age Group	Sampling Point	Cancer Risk Value		Total Cancer Risk Value
		Pb	Cd	
Children	A	0.4548	0.1700	0.6248
	B	0.5542	0.1270	0.6812
	C	0.3805	0.2390	0.6195
	D	0.3808	0.1710	0.5518
	E	0.5874	0.2320	0.8194
	F	0.0302	0.2120	0.2422
Adults	A	0.0133	0.0500	0.0633
	B	0.0162	0.3700	0.3862
	C	0.0120	0.7000	0.7120
	D	0.0110	0.5000	0.5110
	E	0.0020	0.6800	0.6820
	F	0.0009	0.6200	0.6209
Ingestion cancer slope factor (kg day/mg) [47]		8.5 × 10^−6^	3.8 × 10^−4^	

Sites A, B, and C are in the Guu A Parish (upstream), while D, E, and F are in the Westland A parish (downstream).

## Data Availability

Data supporting the conclusions of this study are available upon request from the authors.

## Supplementary Materials

The following supporting information can be downloaded at: https://www.mdpi.com/article/10.3390/jox13040035/s1, Table S1. Pairwise Pearson correlations between the studied physicochemical parameters and potentially toxic elements in water samples from the Pager River, Northern Uganda; Table S2. Eigen-vectors and eigen-analysis of the correlation matrix for the PTEs and physicochemical parameters of water samples from the Pager River, Northern Uganda.
