# Supplementary material for: Health Risks from Intake and Contact with Toxic Metal-Contaminated Water from Pager River, Uganda
Source: J Xenobiot. 2023 Sep 26;13(4):544–59. doi: 10.3390/jox13040035 (PMC10594420; doi:10.3390/jox13040035)
Supplement: Supplementary file 1 [file jox-13-00035-s001.zip › jox-2609403-supplementary.pdf]

Health risks from intake and contact with toxic metal-contaminated water from Pager River, Uganda

Patrick Onen [1], Robin Akemkwene [2], Caroline K. Nakiguli[3], Daniel Nimusiima[3], Daniel H. Ruma [4], Alice V. Khanakwa[5], Christopher Angiro [6], Gadson Bamanya[7], Boniface Opio [8], Allan Gonzaga [7], and Timothy Omara [9,#*]

1 Department of Chemistry, Faculty of Science, University of Kerala, Kariavattom, Thiruvananthapuram 695581, India.
2 Department of Chemistry, Faculty of Education and Humanities, Gulu University, Gulu P. O. Box 166, Uganda.
3 Department of Chemistry, Faculty of Science, Mbarara University of Science and Technology, Mbarara P.O. Box 1410, Uganda.
4 Department of Nutritional Sciences and Dietetics, Kyambogo University, Kampala P.O. Box 1, Uganda.
5 Department of Environmental Health and Disease Prevention, Faculty of Public Health, Lira University, Lira, P. O. Box 1035, Uganda.
6 School of Water, Energy and Environment, Water Science Institute, Cranfield University, College Road, Cranfield MK43 0AL UK.
7 Department of Physical Sciences, Kampala International University, Kampala P.O. Box 20000, Uganda.
8 Department of Science and Vocational Education, Lira University, Lira P.O. Box 1035, Uganda.
9 Chemistry Division, Testing Department, Uganda National Bureau of Standards, Kampala P.O. Box 6329, Uganda.

* Correspondence: prof.timo2018@gmail.com, Timothy.Omara@unbs.go.ug (T.O.)
# Current address: Institute of Chemistry of Renewable Resources, Department of Chemistry, University of Natural Resources and Life Sciences, Vienna (BOKU), Konrad-Lorenz-Str. 24, 3430 Tulln, Austria.

Supplementary materials

Table S1. Pairwise Pearson Correlations between the studied physicochemical parameters and potentially toxic elements in water samples from Pager River, Northern Uganda

Parameters		N	Correlation	95% CI for ρ	P-Value
Temperature	ECD	18	0.017	(-0.453, 0.480)	0.947
pH	ECD	18	0.113	(-0.374, 0.551)	0.655
TDS	ECD	18	-0.204	(-0.613, 0.290)	0.416
Turbidity	ECD	18	0.574	(0.146, 0.821)	0.013*
Cd	ECD	18	-0.320	(-0.685, 0.172)	0.195
Pb	ECD	18	0.136	(-0.353, 0.567)	0.589
pH	Temperature	18	-0.054	(-0.508, 0.423)	0.830
TDS	Temperature	18	0.277	(-0.218, 0.659)	0.266
Turbidity	Temperature	18	-0.192	(-0.605, 0.302)	0.446
Cd	Temperature	18	0.257	(-0.239, 0.646)	0.304
Pb	Temperature	18	-0.067	(-0.518, 0.413)	0.791
TDS	pH	18	-0.357	(-0.706, 0.132)	0.146
Turbidity	pH	18	0.722	(0.385, 0.889)	0.001**
Cd	pH	18	-0.535	(-0.801, -0.090)	0.022*
Pb	pH	18	-0.021	(-0.483, 0.450)	0.934
Turbidity	TDS	18	-0.187	(-0.601, 0.307)	0.457
Cd	TDS	18	0.248	(-0.247, 0.641)	0.320
Pb	TDS	18	0.448	(-0.023, 0.757)	0.062
Cd	Turbidity	18	-0.483	(-0.775, -0.021)	0.042*
Pb	Turbidity	18	0.189	(-0.304, 0.603)	0.452
Pb	Cd	18	-0.121	(-0.556, 0.367)	0.633

Note: ECD = Electrical conductivity, TDS = Total Dissolved Solids. * Significant at the 0.05 level (2-tailed). **Also significant at P<0.01 level (2-tailed).

Table S2. Eigen vectors and Eigen analysis of the correlation matrix for the PTEs and physicochemical parameters of water samples from Pager River, Northern Uganda.

Variable	First Component	Second Component	Third Component
ECD	0.352	0.222	-0.342
Temperature	-0.201	0.152	-0.857
pH	0.485	-0.071	-0.145
TDS	-0.303	0.606	0.005
Turbidity	0.538	0.206	-0.126
Cd	-0.466	-0.061	-0.159
Pb	0.045	0.714	0.294
Initial eigenvalues	2.6392	1.4760	1.0386
Explained variance (%)	37.7	21.1	14.8
Cumulative variance (%)	37.7	58.8	73.6

Note: ECD = Electrical conductivity, TDS = Total Dissolved Solids. * Significant at the 0.05 level (2-tailed).
